# Correction to: TAS2940, A novel brain‐penetrable pan‐ERBB inhibitor, for tumors with *HER2* and *EGFR* aberrations

**DOI:** 10.1111/cas.16081

**Published:** 2024-01-25

Oguchi K, Araki H, Tsuji S, et al. TAS2940, a novel brain‐penetrable pan‐ERBB inhibitor, for tumors with *HER*2 and *EGFR* aberrations. *Cancer Sci*. 2023;114:654–664. doi: 10.1111/cas.15617.

The *p*‐value has been corrected in the following text:

On day 47, tumor volumes decreased, as observed with the NCI‐N87 intracranial model. Survival studies also demonstrated the efficacy of 25.0 mg/kg TAS2940 (Figure 5F, *p* = 0.0271; MST of TAS2940 group, 61 days; MST of control group, 47 days).

In the Supporting Information, Appendix S1, the information about the antibodies used in experiment is insufficient and has been updated in the following sentences in the “Antibodies” section on page 1:

Line 5: anti‐HER2 (Cat# 4290, #2165 only for poziotinib)

Line 7–8: anti‐AKT (Cat# 4691, #4685 only for poziotinib)

Line 8–9: anti‐cleaved PARP (Cat# 5625, #9541 only for poziotinib)

We apologize for these errors.

